# Contributions to Therapeutics

**Published:** 1845-05

**Authors:** J. Moore Neligan

**Affiliations:** Physician to Jervis-street Hospital, Lecturer on Materia Medica and Therapeutics in the Dublin School of Medicine, &c.


### PRACTICAL MEDICINE, &c.

*Contributions to Therapeutics.* By J. Moore Neligan, M. D., Physician to Jervis-street Hospital, Lecturer on Materia Medica and Therapeutics in the Dublin School of Medicine, &c.

### ON THE EMPLOYMENT OF CONIUM IN PAINFUL DISEASES.

In the following communication it is my intention to offer a few practical observations on the anodyne and sedative powers of the common hemlock, and to illustrate its medicinal properties by relating a few cases in which its employment has been attended with much benefit. Although much employed and highly extolled by the ancients, hemlock had fallen into complete disuse in modern medicine, until the latter end of last century, when it was again introduced, and generally used, owing to the high terms in which it was spoken of By Baron Störk, who, in 1762, published an account of the physiological and therapeutical properties of this drug. Störk ascribed two distinct therapeutical properties to the preparations of hemlock; first, that of a powerful anodyne and sedative, and second, that of a deobstruent and alterative, especially in the treatment of glandular or visceral enlargements, of scrofulous affections, of secondary syphilis, and of chronic cutaneous diseases. In the present day, but little faith is placed in the deobstruent virtues of the drug, and much difference of opinion exists even as to its anodyne properties, consequently it has again lost much of its reputation as a medicine, and is not nearly so much employed as it deserves to be.

Since the discovery of the active principle of the plant, this almost universal discredit of its medicinal powers has been very satisfactorily accounted for, as it has been distinctly proved, that the application of even a moderate degree of heat, when continued for any time, causes it to undergo decomposition, and therefore that the extract, (the preparation most generally employed) when prepared in accordance with the directions of the Dublin and London Pharmacopœias, is for the most part, inert, or nearly so; that this is the case I have repeatedly satisfied myself, by applying the potash test to various samples of the extract of our Pharmacopœa, obtained at the best shops. The potash test is of so simple a character, so easy in its application, and so certain in its results, that we should never omit its employment before commencing the use of any of the preparations of hemlock. It consists merely in triturating in a mortar the preparation we wish to test with a small quantity of strong caustic potash, when the peculiar odor of the active principle, *conia*, is in a few moments emitted; care, however must be taken, not to confound this odor with that of the plant itself, from which it differs most remarkably, the latter bearing a singular resemblance to the smell of mice, while that of conia is a peculiar, penetrating, very disagreeable, somewhat alkaline odor, an acquaintance with which may be easily acquired by applying the test to the fresh green leaves, or to the recently gathered ripe fruit.

In commencing, then, any new investigation into the medicinal action and uses of hemlock, it becomes of much importance to take especial care that the preparations of the drug which we administer, should have their energy unimpaired, and the peculiar properties which exist in the recent plant as little changed as possible. The preparation which I employed in the following cases, and which I have been in the habit of prescribing for the last two years, under the name of Succus Conii, is simply prepared as follows: Take of fresh hemlock leaves any quantity, express the juice in a tincture press, set it aside for forty-eight hours, pour off the clear, supernatant liquor, from the fecula and chlorophylle which it has deposited, and lastly, add to it a fifth part, by measure, of rectified spirit. This preparation I have found to keep well for two years, and its uniform strength, as well as the facility with which we can increase or diminish the dose we are administering, gives it a decided advantage over either the extract or powder of the fruit or leaves. The best time for gathering the leaves is when the plant is in full flower, and previous to submitting them to expression the stalks should be carefully picked out and rejected, the leafy part alone being used. As in many instances it is often of great advantage to possess an active preparation of a remedy in a solid state, I have tried many ways of preparing an extract of hemlock, which would retain unimpaired the medicinal powers of the plant, and the best I find is to be obtained by submitting the expressed juice, prepared as above to spontaneous evaporation; but even this extract, no matter how well and carefully preserved, soon looses all traces of *conia*.

Hemlock, when administered in medicinal doses to an individual laboring under disease, appears to me to produce its beneficial effects by allaying nervous excitability, and diminishing muscular pain; under its use also, both the force and frequency of the heart's action are lowered, but in no instances have I seen the least tendency to drowsiness or sleep. This is quite consonant with the account given by Christison of the action of hemlock when its poisonous effects are produced; "that it does not excite convulsive spasms or bring on insensibility, but that it exhausts the nervous energy of the spinal chord and voluntary muscles, occasioning merely convulsive tremors and slight twitches, and eventually, general paralysis of the muscles, and consequent stoppage of the breathing." The active principle, *conia*, according to the same able authority, produces a similar remarkable action on the spinal chord, "a few drops killing a small animal, such as a rabbit, cat, or puppy, in a few minutes, causing general paralysis, slight convulsive tremors, and death from the suspension of the breathing, without any alteration in the appearance of the blood." Such being the effects of hemlock, and its alkaloid, when given in poisonous doses, it can be readily understood that when administered as a medicine it will produce no very apparent physiological action, and that, in producing beneficial results, it appears to act insensibly on the system. The only manifest effect which I have seen it produce is where its use has been persevered in for some time, or the doses rapidly increased, when the patient generally complains of a disagreeable sensation of dryness of the throat, with a feeling of constriction and difficulty of swallowing, amounting to actual pain, and which always compels us either to suspend the use of the medicine altogether for a few days, or greatly to diminish the dose in which it has been given.

The diseases in which I have administered hemlock with decided advantage are rheumatic affections, both subacute and chronic, particularly when attended with severe pain, neuralgia, and senile gangrene. And although I have employed it very extensively, both in hospital and private practice in those diseases, I have met with but very few instances in which this remedy failed to afford relief: nevertheless, some cases occasionally occur, in which, as is the case with most other medicines, it does not appear to produce the least benefit.—*Dublin Jour.* in *Med. Examiner.*

OBSTETRICS AND DISEASES OF FEMALES.

*On Detaching the Placenta in cases of Placenta Prævia.* By Dr. RADFORD, M. D.—Since my observation on galvanism in uterine hæmorrhage, published in the *Provincial Medical and Surgical Journal*, I have had letters from many gentlemen, inquiring whether I confirmed the practice of detaching the placenta in cases of placenta prævia to those of exhaustion alone. In order, then, to supersede the necessity of writing to each correspondent, I make the reply through the same channel. In my paper I stated that I had detached the placenta in a case which occurred in 1819, but did not then state that it was unattended by exhaustion. From this and other cases then alluded to, I conclude, that on a complete separation of the placenta, the hæmorrhage is immediately and completely suppressed, provided the uterus is in a condition to so far contract, as to force down the head with the placenta upon the uterine openings. By this practice it may be said that the life of the child is sacrificed: but this will not always happen. We find from hospital and individual reports, that the child is usually dead when the case has been treated by the present recognized means.

In nearly all the cases which I have collected and referred to in my paper, of expulsion of the placenta by the natural efforts, we find that the mother recovered; and when this fortunate event did not happen, it depended upon the serious impression made upon the vital powers before the placenta was completely detached.

It may also be stated that uterine phlebitis takes place more frequently in cases of placenta prævia, when the ordinary practice is adopted, than we observe in the same number of cases of accidental hæmorrhage. This result, in the opinion of the writer, arises from the contusion and slight lacerations which are consequent upon a forced delivery.

From the above statement, I consider I am justified in recommending a modified practice; but I shall not enter fully into the details of the plan, as this brief communication will not allow of it.

First. Then, as neither delivery, nor detaching the placenta, ought ever to be attempted until the cervix and os uteri will safely allow of the introduction of the hand, rest, the application of cold, but, above all, the use of the plug, must never be omitted in cases where they are respectively required.

Secondly. If there are unequivocal signs of the child's death, the placenta is to be completely detached, and the membranes are to be ruptured. The case is then to be left to the natural efforts, provided there be sufficient uterine energy; if otherwise, the ordinary means are to be used, and, in addition, galvanism.

Thirdly. When the narrow pelvis exists in connection with placenta prævia, the practice is to detach the placenta and to remove it, then to perforate the head as soon as the condition of the parts allows, and to extract it by means of the crotchet.

Fourthly. When the os uteri is partially dilated, and dilatable so as to admit of the easy introduction of the hand, when the membranes are ruptured, and strong uterine contraction exists, the practice is to detach the placenta completely.

Fifthly. In all cases of exhaustion, as already referred to in my paper, the practice is to draw off the liquor amnii, by perforating the placenta, as then recommended, then to detach completely this organ, and apply galvanism.

Sixthly. In all cases of partial presentation of the placenta, the artificial rupture of the membranes will generally be found sufficient to arrest the hæmorrhage, but if that should prove ineffectual, then we must apply galvanism.

The practice of removing and detaching the placenta was adopted by some of the older writers; and as I have mentioned in my paper "On galvanism applied to the treatment of uterine hæmorrhage," I detached this organ in the year 1819, although it was not my custom to do so.

Early in October, I received a letter from my respected friend, Professor Simpson, in which he stated he removed the placenta in a case of unavoidable hæmorrhage. He "had the placenta on a plate two hours before the baby was born." The mother recovered. Dr. Simpson has collected a great number of cases of expulsion of the placenta before the child, and has come to the conclusion, that the practice of its removal, in some cases of placenta prævia, is calculated to save the parent's life.

I am glad to have my views on this most important subject corroborated by an authority so deservedly esteemed as Dr. Simpson, to whom I am disposed to award every degree of merit which really belongs to him, as having by observation and research accumulated materials to bring him to the same conclusion at which I arrived myself. Although I feel thus gratified in having the authority of Dr. Simpson in support of this practice, I must confess that it is to be the late Mr. Kinder Wood, who for many years was an active and deservedly respected colleague oi mine at the Lying-in Hospital, that the merit (if there be any) is due for first, as a modern obstetrician adopting this practice, and also recommending it in his lectures. The practice I allude to, is that of detaching and removing the placenta in cases of unavoidable hæmorrhage, attended with exhaustion. In the foregoing observations I have ventured to recommend this practice as applicable to cases in which there exist different conditions, convinced that there are many mothers sacrificed by the rash manœuvres consequent on a forced and indiscriminate delivery.—*Provin. Med. and Surg. Jour.* in *N. Y. Jour of Med.*

